# Supplementary material for: From Laboratory towards Industrial Operation: Biomarkers for Acidophilic Metabolic Activity in Bioleaching Systems
Source: Genes (Basel). 2021 Mar 25;12(4):474. doi: 10.3390/genes12040474 (PMC8065656; doi:10.3390/genes12040474)
Supplement: Supplementary file 1 [file genes-12-00474-s001.pdf]

From laboratory towards industrial operation: biomarkers for acidophilic metabolic activity in bioleaching systems

8. Supplementary Materials

Table S1. Primers designed for *At. thiooxidans* (ATT), *At. ferrooxidans* (AFE), *L. ferriphilum* (LII), and *Sulfobacillus sp* CBAR13 (STO) and used for real time quantitative PCR (RT-qPCR) analysis.

Gene	Primer	Sequence (5' to 3')	Efficiency (E)	Annealing T° (°C)
rbcL1	ATT-rbcL-1 F<br>ATT-rbcL-1 R	TCAAGGAGTACCGCCACACAT<br>TAAGCAGGTCAGTCCAGACCGT	1.9	60
rbcL2	ATT-rbcL-2 F<br>ATT-rbcL-2 R	ATACACTGCTTTCGTGCTGGC<br>CAGAAATGATCGGTGTAGTGGG	1.9	60
csoS2	ATT-csoS2 F<br>ATT-csoS2 R	TATCTGGTACGCAACTGGGC<br>CCATCGCCACTTTCTGAGGT	1.9	60
csoS3	ATT-csoS3 F<br>ATT-csoS3 R	ACCAATGCCACCATGCTTTG<br>AGCCTGCTCGGTAAAGTCAC	1.9	60
csoS4	ATT-csoS4 F<br>ATT-csoS4 R	CTACGGGTGTTGTCTGATGC<br>GCCGAACCTGCTACCGTAAAC	1.9	60
16S	ATT-16S F<br>ATT-16S R	GGGTGCTAATANCGCCTGCT<br>TTGTGCGGGCCCCCGTCAAT	1.9	60
gyrA	ATT-gyrA F<br>ATT-gyrA R	GCTTTGTTCGATCTGGTGCCT<br>ACCACTGCGGTATGCTTCAA	1.8	60
rbcL-1	AFE-rbcL-1 F<br>AFE-rbcL-1 R	AATCTTTGGCGATGATGCGGTA<br>GGACTGTCTTTCGCTGCGTT	2.0	60
rbcL-2	AFE-rbcL-2 F<br>AFE-rbcL-2 R	CTACTACAAGGGGCGCGCATAC<br>CGTTACCAACCAGCGAGGTG	2.0	60
alaS	AFE-alaS F<br>AFE-alaS R	CGGAGTACCGGTATTGATCA<br>CGTCCAGTCATTCCTTTACC	1.9	60
treZ	LII-treZ F<br>LII-treZ R	GAAACCCGGCCGTTTCTCTA<br>GAACGCGGCGAACTCTTTTT	2.0	60
treY	LII-treY F<br>LII-treY R	GATCAGCCTTCCGATCCTCG<br>CTCGAGTTCCCGGTTTTCCA	2.1	60
treX	LII-treX F<br>LII-treX R	GGTCGGAGAAAATCCCCGTT<br>TGCCGCATGTTCAGGGTATT	2.3	60
lamB	LII-lamB F<br>LII-lamB R	TGAACAGTTCGGCCTTCTCC<br>AGCAGAACTTCCGGTTCCAC	1.9	60
galU	LII-galU F<br>LII-galU R	TTTTGAAAGCCACGGTGCAG<br>TTTCCGCGGATGGAGAAGTC	2.0	60
gadAB	LII-gadAB F<br>LII-gadAB R	GATGATTCGCTTTCCGCCAC<br>GGTTTCCGTCGAGTTCCAGT	2.0	60
nifH	LII-nifH F<br>LII-nifH R	TGCGACCCCAAGGCTGATTC<br>GCCCTCCAGATTCCACGCAA	2.1	60
amt2	LII-amt F<br>LII-amt R	CCGTGGATCTCGATGAACAT<br>ACCACGATATTCAGGGCGAT	2.0	60
glnA	LII-glnA F<br>LII glnA R	GGAAGTGCATCATCATGAGG<br>CATATCCGCCTTTCTGGAAG	1.9	60
glnB	LII glnB F<br>LII glnB R	GTCTCGGGCTACACGATCAT<br>AACACCACACCGGAATACCG	2.0	60
alaS	LII-alaS F<br>LII-alaS R	TACCCGGAGCTTAGAACATC<br>TATCGAGCGGAAATCCATGC	2.0	60
16S	LII-16S F<br>LII-16S R	TACAAGCTTCCGCTCCTG<br>CCGGGCAAAAGTGGTTTACA	1.8	60
carb	STO-carb F<br>STO-carb R	ATGGCGTCGTGTCTCACTTT<br>GCGGCAAAACGGTCTTGTAA	1.9	60
cytbdl	STO-cytbdl F<br>STO-cytbdl R	CTCCTGGGTCGCAAGTCATT<br>TGTAGGATCCCCTCCCGTTT	2.0	60
sulf	STO-sulf F<br>STO-sulf R	GCAAGTTCATCCCGCTTCAA<br>TAACCGACCGGAATGGTCAC	1.9	60
chypll	STO-chypll F<br>STO-chypll R	CTTGGCCCACGTTTCATTCG<br>TGGGTAGTCGGACACTTCCA	2.0	60
tehy	STO-tehy F<br>STO-tehy R	AGTCCTTTTGCACGCGTAGT<br>ACTTCGAAAGCCGCATTGTG	2.0	60
fadp	STO-fadp F<br>STO-fadp R	GGCAACAACGTCAGCTTCAA<br>CCAATCGCCACACATTACGC	2.0	60
gyrA	STO-gyrA F*<br>STO-gyrA R*	CGGATTTACCGGGTCAAGGT<br>ATGCGCTCATCAGCTTCCAT	2.0	60

Table S2. RNA quantity and quality obtained from bioleaching column test and industrial bioleaching strip samples.

Samples	RNA (ng/uL) (*range)	260/230 ratio (*range)	260/230 ratio (*range)
Col 1.: control	76 - 146*	1.8 - 2.0*	0.7 - 0.9*
Col 2.: O_2 and CO_2 availability	48 - 160*	1.7 - 2.0*	0.4 - 2.2*
Col 3.: N_2 and NH_4^+ availability	52 - 114*	1.8 - 2.0*	0.3 - 1.9*
Col 4.: osmotic stress (SO_4^{2-} impurity)	66 - 131*	1.7 - 2.0*	0.7 - 1.2*
S-410-W0-D	30.1	1.55	0.7
S-410-W0-M	49.4	1.78	1.5
S-410-W2-D	26.5	1.68	1.1
S-410-W2-M	7.8	1.77	1.4
S-410-W4-D	173	1.64	0.7
S-410-W4-M	47.6	1.61	1.1
S-132-D	317.8	-	-
S-327-D	184.6	-	-
S-413-D	36.9	-	-
S-414-D	106.8	-	-
S-405-D	133	1.9	1.7
S-405-B	109	1.9	0.4
S-405-M	153	2.0	0.4
S-405-D	133	1.9	1.7
S-405-B	109	1.9	0.4
S-405-M	153	2.0	0.4

Table S3. Estimated *p-values* for multiple regression models of the *At. thiooxidans*, *L. ferriphilum* IESL-25, and *L. ferriphilum* type strain DSM 14647 growing at different CO_2 and SO_4^{2-} levels.

Strain	Response	Transformation	p values								Regression Equation
			Continuous predictors				Interactions		Categorical variables		
			Time (h)	Time (h) * Time (h)	SO_4^{2-} (g L^{-1})	CO_2 (ppm)	Time (h) * SO_4^{2-} (g L^{-1}) (Time(h) * $[SO_4^{2-}]$)	Time (h) * CO_2 (g L^{-1}) (Time(h) * $[CO_2]$)	SO_4^{2-} (H, M, L)*	CO_2 (H, L)**	
At. thiooxidans IESL-33 (Fig. 2)	Cell number (cell mL^{-1})	square root	<0.0001					<0.0001		0.049	L cel mL-1^0.5 = 14784273 + 260633 Time (h)<br>H cel mL-1^0.5 = 10426501 + 429367 Time (h)
	pH		<0.0001							<0.01	L pH = 2.8462 - 0.00946 Time (h)<br>H pH = 2.7537 - 0.00946 Time (h)
L. ferriphilum DSM 14647 (Fig. 7)	log_{10} (Cell number (cell mL^{-1}))	square root	<0.0001	0.001	<0.0001		0.006				2.50 log cell mL-1^0.5 = 2.5661 + 0.00986 Time (h) - 0.000092 Time $(h)^2$<br>25.00 log cell mL-1^0.5 = 2.3832 + 0.01231 Time (h) - 0.000092 Time $(h)^2$<br>50.00 log cell mL-1^0.5 = 2.4076 + 0.01203 Time (h) - 0.000092 Time $(h)^2$
	Fe II (g L^{-1})		<0.0001		0.936		(=0.02)				L Fe+2 (g/L) = 3.461 - 0.07799 Time (h)<br>M Fe+2 (g/L) = 3.540 - 0.06888 Time (h)<br>H Fe+2 (g/L) = 3.444 - 0.05642 Time (h)
L. ferriphilum IESL-25 (Fig. 8)	log_{10} (Cell number (cell mL^{-1}))	square root	<0.0001	0.012							log cell mL-1^0.5 = 2.55066 + 0.009258 Time (h) - 0.000093 SO42- 0.000048 Time $(h)^2$
	Fe II (g L^{-1})		<0.0001				(<0.0001)		0.025		L Fe 2+ = 3.413 - 0.08750 Time (h)<br>M Fe 2+ = 4.020 - 0.08743 Time (h)<br>H Fe 2+ = 3.679 - 0.05548 Time (h)

* L, M and H represent SO42- concentration of 2.5, 25 and 50 g L-1, respectively.

** L and H represent CO2 levels of 50 and 500 ppm, respectively

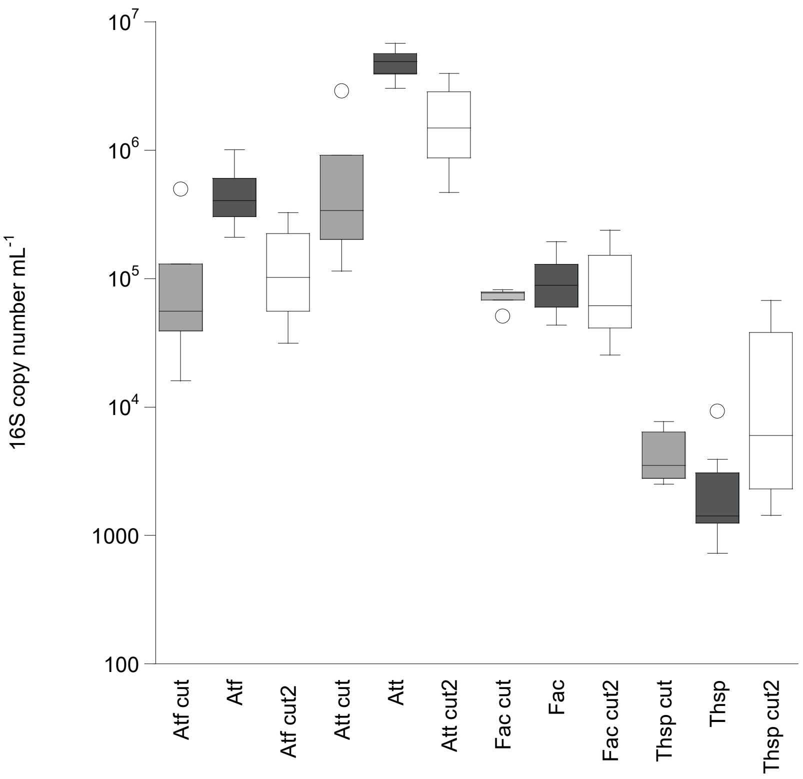

Figure S1. Copy numbers of 16S rRNA by real-time PCR analysis of autotrophic (Atf and Att) and heterotrophic (Fac and Thsp) microorganisms in column experiment with on/off air feeding. Cut = air feeding interrupted. Atf: *Acidithiobacillus ferrooxidans*; Att: *Acdithiobacillus thiooxidans*; Fac: *Ferroplasma acidiphilum*; Thsp: *Thermoplasma* sp.

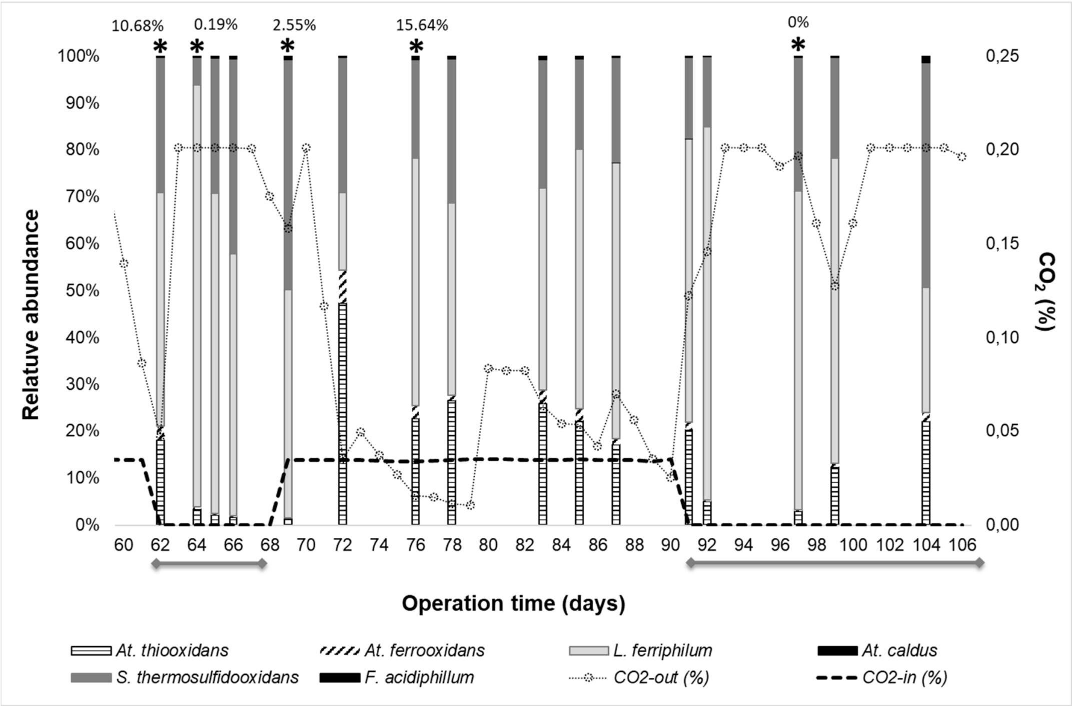

Figure S2. Characterization of col.2 community in response to input-airflow interventions. Samples used in the transcriptional analysis are indicated with an asterisk and their O_2 levels (%). Horizontal bars on the below the x-axis indicate the two periods when input-airflow was inter-rupted in the column (days 62 to 68 and days 91 to 104). The input (CO_2-in) and output (CO_2-out) CO_2 levels are shown.

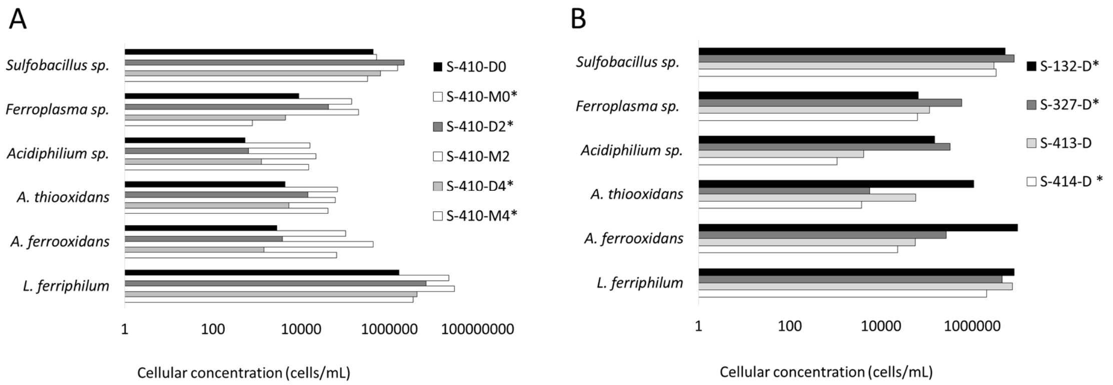

Figure S3. Community characterization of the industrial strips S-410 (A) and S132, 327, 413, 414 (B) of MEL bioleaching heap responding to aeration interventions. Samples that were used in the transcriptional analysis are indicated with an asterisk (*). A) 0, 2, 4 correspond to the three samplings performed biweekly before (0) and after (2, 4) aeration intervention in strip 410. M are samples taken on the base of the fourth lift. D is a drop (base of the first lift).

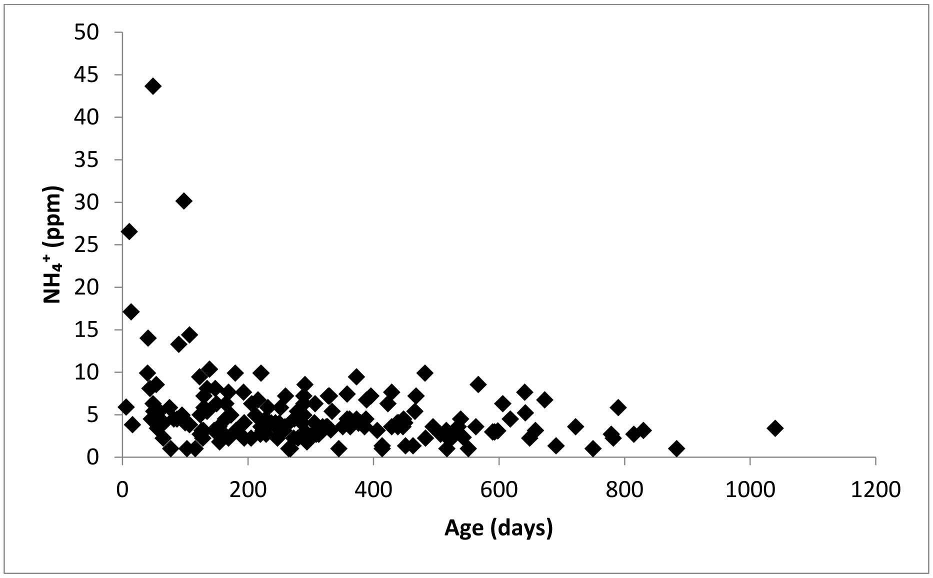

Figure S4. Ammonium level measured in industrial PLS solutions vs. the age of the heap expressed as the day after the start of irrigation.
